# Acute Thromboembolism from Trauma in a Patient with Abdominal Aortic Aneurysm

**DOI:** 10.5811/cpcem.2021.4.52137

**Published:** 2021-07-21

**Authors:** Solomon Sebt, Chris Kim, Wirachin Hoonpongsimanont, Eric Leroux

**Affiliations:** Eisenhower Medical Center, Department of Emergency Medicine, Rancho Mirage, California

**Keywords:** Abdominal aortic aneurysm, acute thrombosis, embolectomy, acute limb ischemia

## Abstract

**Case Presentation:**

A 64-year-old man with a history of a 5.5-centimeter (cm) abdominal aortic aneurysm (AAA) presented to the emergency department (ED) complaining of severe back pain after climbing over a fence and falling a distance of eight feet. Prior to arrival, the prehospital paramedics reported that the patient did not have palpable pulses in either lower extremity. The initial physical examination in the ED was significant for absent dorsalis pedis pulses bilaterally as well as absent posterior tibialis pulses bilaterally and cold, insensate lower extremities. Point-of-care ultrasound identified an approximate 7-cm infrarenal AAA with a mural thrombus present. After receiving several computed tomography (CT) studies including CT head without contrast and CT angiography of the chest, abdomen and pelvis, the patient was diagnosed with acute thrombosis of AAA and associated thromboembolic occlusion of both his right and left distal iliac vessels causing bilateral acute limb ischemia. He immediately received unfractionated heparin and was admitted to the hospital for embolectomy and intra-arterial tissue plasminogen activator.

**Discussion:**

Acute thrombosis of AAA and subsequent thromboembolic events are a rare but significant complication that can occur in patients with a history of AAA. Thromboembolic events may occur spontaneously or in the setting of blunt abdominal trauma. Common presenting signs and symptoms include distal limb ischemia and absent femoral pulses. Timely management and recognition of this rare complication is vital as this condition can ultimately result in limb loss or death if not treated in a timely manner. Heparinization after confirmation of non-ruptured AAA as well as vascular surgery, and therapeutic and vascular interventional radiology consultations are key steps that should be taken to decrease patient morbidity and mortality.

## CASE PRESENTATION

**Image 1:**
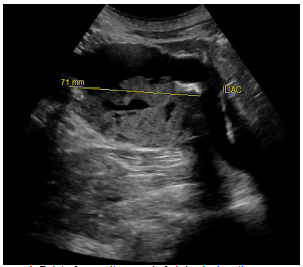
Point-of-care ultrasound of abdominal aortic aneurysm using 3.5 megahertz probe in transverse view above the level of bifurcation of iliac vessels, showing aneurysmal dilatation of 7.1 centimeters (71 mm). Associated mural thrombus within the lumen of the aorta can be visualized.

**Image 2:**
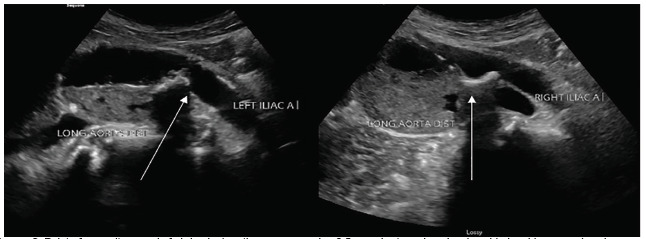
Point-of-care ultrasound of abdominal aortic aneurysm using 3.5 megahertz probe, showing side-by-side comparison in transverse view of the aorta, at the level of bifurcation of the left and right iliac arteries as labeled. Dislodged clot can be seen at the level of bifurcation shown above (arrows).

A 64-year-old male was brought to the emergency department (ED) by helicopter after sustaining an injury falling over a fence. The patient reported injuring his “stomach” and feeling a pop followed by severe back pain. On scene, the prehospital paramedics reported no pulses in the bilateral lower extremities. Prior to arrival, the patient had been given 250 micrograms fentanyl, 20 milligrams (mg) labetalol, and 4 mg of midazolam by medics for concern of possible aortic dissection. Vital signs upon arrival included a blood pressure of 166/102 millimeters of mercury, heart rate of 92 beats per minute, 16 respirations per minute, an oxygen saturation of 96% on room air, and a temperature of 99.2ºF. Physical examination was notable for 2+ carotid and radial pulses bilaterally, and absent dorsalis pedis and popliteal pulses bilaterally. Point-of-care ultrasound identified a large infrarenal abdominal aortic aneurysm (AAA) of approximately 7 centimeters (cm) in size with contained thrombus within the lumen. (Images 1 and 2).

Computed tomography angiography of the chest, abdomen, and pelvis reaffirmed the presence of an acutely ulcerated thrombus contained within the lumen of the aorta and no evidence of extraluminal contrast extravasation. This information, combined with vascular duplex ultrasound of the lower extremity confirming extensive clot burden down to the level of the dorsalis pedis, was presented to our vascular surgeon and therapeutic interventional radiologist on call. In shared decision-making with the patient, this multidisciplinary team initiated anticoagulation with unfractionated heparin and transferred the patient to the operating room for emergent embolectomy and intra-arterial tissue plasminogen activator.

## DISCUSSION

Our patient was ultimately diagnosed with acute thromboembolism of AAA. The incidence of this type of mechanistic complication is rare, occurring in approximately 0.7–2.8% of surgically managed cases.^1^ The most common signs and symptoms of acutely thrombosed AAA include acute lower limb ischemia and absent femoral pulses, which are reported in approximately 45.7% and 68.6% of the time in reported cases, respectively.^2^ Timely management of these patients is paramount as the negative outcomes in these scenarios can often include limb loss or even death.^2^ Acute thrombosis of AAA has been reported to occur at various aneurysm sizes ranging from 3.5–10.5 cm.^3^ It is proposed that systemic heparinization immediately after diagnosis and prompt surgical revascularization can reduce the mortality rate.^4^

**CPC-EM Capsule**

What do we already know about this clinical entity?

*Acute thrombosis of abdominal aortic aneurysms (AAA) and subsequent thromboembolic events are rare complications, particularly in the setting of blunt abdominal trauma*.

What is the major impact of the image(s)?

*Rapid diagnosis aided by point-of-care ultrasound and angiography can identify acute aortic thrombosis and help minimize severe morbidity secondary to limb ischemia*.

How might this improve emergency medicine practice?

*Recognizing acute thrombosis of an AAA in the setting of trauma and intervening promptly has significant implications on patient morbidity and mortality*.

Various mechanisms behind acute thrombosis of AAA have been discussed in the literature, including acute low-flow state due to occlusion, hypercoagulation disorder or hypercoagulability secondary to neoplastic disease, cardioaortic embolization due to cardiac arrhythmias, dislocation of a fragment of the mural thrombus within the aneurysm sac secondary to trauma or spontaneously, and hypotension and low-flow state secondary to other causes.^2^,^4^,^5^ Our patient likely had a dislocation/rupture of his mural thrombus within the aneurysmal sac secondary to traumatic abdominal impact leading to distal occlusion. Moreover, he was treated with heparin after verifying absence of aortic rupture and admitted to the hospital for further treatment with embolectomy. Unfortunately, due to the patient’s extensive clot burden, he ultimately required bilateral below knee amputations despite aggressive attempts at pharmacological and interventional management.

Comprehensive care of patients with AAA complication in the ED is facilitated through a combination of a thorough physical examination, appropriate radiographic studies, and consultations with specialists. Recognizing these thromboembolic events and ensuring that both surgical and interventional candidates are promptly identified has significant implication on long-term morbidity and mortality.
